# Determinants of Adherence to Antihypertension Medications Among Patients at a Tertiary Care Hospital in Islamabad, Pakistan, 2019

**DOI:** 10.5888/pcd20.220231

**Published:** 2023-05-25

**Authors:** Nadia Noreen, Faiza Bashir, Abdul Wali Khan, Malik Muhammad Safi, Waheed Ahmad Lashari, Dagmara Hering

**Affiliations:** 1 Directorate of Central Health Establishments, Islamabad, Pakistan; 2 Pakistan Health Research Council, National Institute of Health, Islamabad, Pakistan; 3 Ministry of National Health Services, Khosar Block, Islamabad, Pakistan; 4 Expanded Program of Immunization, Baluchistan Department of Health, Pakistan; 5 Department of Hypertension and Diabetology, Medical University of Gdansk, Gdansk, Poland

## Abstract

**Introduction:**

Medication nonadherence leads to poor health outcomes, frequent complications, and high economic impact. Our objective was to assess the determinants of adherence to medication regimens among patients with hypertension.

**Methods:**

We conducted a cross-sectional study of patients with hypertension attending the cardiology clinic of a tertiary care hospital in Islamabad, Pakistan. Data were collected by using semistructured questionnaires. A score of 7 or 8 on the 8-item Morisky Medication Adherence Scale was classified as good adherence, 6 as moderate, and less than 6 as nonadherence. Logistic regression was performed to determine covariates associated with medication adherence.

**Results:**

We enrolled 450 patients with hypertension (mean age, 54.5 y; SD, 10.6). Medication adherence was good among 115 (25.6%) patients and moderate among 165 (36.7%); 170 (37.8%) patients were nonadherent. Most patients (72.7%) had uncontrolled hypertension. Nearly half (49.6%) were unable to afford monthly medication. In bivariate analysis, nonadherence was associated with female sex (odds ratio [OR], 1.44; *P* = .003) and long waiting times in the health care facility (OR, 2.93; *P* = .005*)*; the presence of comorbidities (OR, 0.62; *P* = .01) was associated with good adherence. In multivariate analysis, nonadherence was associated with unaffordability of treatment (OR, 2.25; *P* = .002) and uncontrolled hypertension (OR, 3.16; *P* < .001). Good adherence determinants included adequate counseling (OR, 0.29; *P* < .001) and education (OR, 0.61; *P* = .02).

**Conclusion:**

Addressing identified barriers, including medication affordability and patient counseling, should be included in Pakistan’s national policy on noncommunicable disease.

**Summary**

**What is already known on this topic?**

The prevalence of uncontrolled hypertension in Pakistan is high. Improving medication adherence reduces the burden of cardiovascular disease.

**What is added by this study?**

Unaffordability, female sex, and long waiting times in the health care facility were predictors of nonadherence to antihypertension medicines. Effective counseling and state-covered medical care were associated with improved medication adherence.

**What are the implications for public health practice?**

An action plan with targeted interventions and policies is needed to promote optimal medication compliance within the Pakistan National Plan for the prevention and management of hypertension.

## Introduction

Higher prevalence of hypertension in low- and middle-income countries (LMICs) drives the global burden of cardiovascular disease (CVD) (1). Pakistan is the fifth most populous country in the world, and approximately half of its population has at least 1 chronic disease (2). The prevalence of hypertension has been estimated at 26.7% in Pakistan (3). Although few data are available on hypertension prevalence in Pakistan and its control rate, of the 50% of adults diagnosed with hypertension, only half were treated and only 12.5% of were able to control their blood pressure (4). The growing incidence of uncontrolled hypertension increases the burden of noncommunicable diseases, with an estimated 3.87 million resultant premature deaths projected in Pakistan by 2025 (2). The economic burden of deaths from these diseases in Pakistan is estimated to double from $152 million in 2010 to $296 million in 2025 (2).

The Pakistani government has one of the lowest health sector budgets among LMICs (5). The availability and affordability of blood pressure–control medicines and accurate blood pressure measuring devices are substantial barriers to proper hypertension management in Pakistan (6). Medication nonadherence leads to poor health outcomes and increased health care costs. Patient nonadherence and clinical inertia negatively affect hypertension management (7).

Information is scarce on adherence to antihypertension medications in Pakistan. Previous studies were limited in their sample size (8,9). No previous studies in Pakistan have evaluated medication adherence in a large cohort of hypertensive patients linked to associated determinants. Our aim was to assess the determinants of patient adherence to antihypertension medications.

## Methods

### Study design, setting, and population

We conducted a hospital-based cross-sectional study of adult hypertensive patients attending the cardiology clinic of the Federal Government Polyclinic Hospital in Islamabad Capital Territory, Pakistan, from July through October 2019. The hospital is a government-run, tertiary care facility with a catchment area that includes the capital city of Pakistan and surrounding areas. On average, 150 to 200 outpatients with various cardiovascular diseases attend the cardiology clinic daily. Free and low-cost services at this facility attract a large number of patients. We selected the Federal Government Polyclinic Hospital to increase the reliability of study results and ensure generalization of the data to the Pakistani population. Patients were included if they were 1) aged 18 years or older, 2) had been visiting a cardiology clinic for the last 6 months, and 3) were previously diagnosed with hypertension. The exclusion criteria were 1) newly diagnosed patients with hypertension at the first visit to the hospital and 2) patients with a critical illness or cognitive impairment.

### Operational definitions

Adherence was defined according to the World Health Organization as the degree to which a person's behavior, including taking medication, corresponds with agreed recommendations from a health care provider (10). This includes the initiation of the treatment, implementation of the prescribed regimen, and discontinuation of the pharmacotherapy.

Nonadherence has been classified as primary and secondary. Primary nonadherence is defined as the frequency at which patients fail to fill prescriptions when new medications are started. Secondary nonadherence is defined as filling prescriptions but not taking the medication as prescribed. Grade I hypertension was defined as blood pressure of 140 to 159 mm Hg systolic or 90 to 99 mm Hg diastolic. Uncontrolled hypertension was defined as systolic or diastolic blood pressure 140/90 mm Hg or higher.

### Sample size, assumption, and sampling procedure

The study sample, based on an unknown adherence prevalence of 50%, was calculated with a 95% confidence limit, 5% margin of error, and 10% nonresponse rate for a sample size of 450. To select participants, we used a systematic random sampling technique of every third patient who met our inclusion criteria.

### Variables of interest

The outcome variable was adherence to a hypertension medication regimen. Independent variables were level of satisfaction with physician and medicine affordability, accessibility, efficacy, and tolerability. Variables that may influence medication adherence were grouped into 1) patient-related factors (age, sex, marital status, level of education, and income); 2) services-related factors (access to health care, prescription patterns of clinicians, and adherence counseling); 3) disease-related factors (duration of hypertension; knowledge of and behavior toward hypertension, its prevention, treatment, associated comorbidity, disease-associated complications, and hospitalizations); and 4) medication-related factors (adverse effects, type of drug therapy, dosage frequency).

We used the self-reported 8-item Morisky Medication Adherence Scale (MMAS-8) questionnaire to measure adherence to hypertension medication regimen. MMAS-8 was scored as 1 point for each no answer and 0 points for each yes. The total score ranged from 0 points (completely nonadherent) to 8 points (completely adherent). An MMAS-8 score of 7 or 8 was classified as good, a score of 6 as moderate, and a score less than 6 as nonadherent.

### Data collection

We collected data by using a semistructured pretested questionnaire (in both English and Urdu) administered by a team of trained data collectors. These questionnaires included both closed-ended and open-ended questions based on an extensive literature review focused on the variables of interest that may influence adherence to antihypertension medication. MMAS-8 was used to measure medication adherence. We used the modified Kuppuswamy scale to measure the socioeconomic status of respondents, taking into account a composite score including the education and occupation of the family head and monthly household income, with a score of 1 to 29. The monthly income and average monthly cost for hypertension treatment provided by respondents were converted from Pakistani rupees to US dollars at the daily calculation rate by using a currency converter (www.forex.pk) during data collection.

Before interviewing the patient, the trained data collectors took 2 blood pressure readings by using a manually calibrated mercury sphygmomanometer and stethoscope with an appropriately sized cuff to ensure accuracy. This method is the most conventional method used in Pakistan and is considered the gold standard according to the third National Hypertension Guidelines, Pakistan Hypertension League 2018 (11). To ensure consistency among participants, the patient was in a sitting position with a cuff placed on the right arm at heart level and blood pressure was measured in a 1-minute interval after 5 minutes of rest before calculating the average blood pressure (11).

### Statistical analysis

We used SPSS version 23 (IBM Corp) and Epi Info Version 7 (Centers for Disease Control and Prevention [CDC]) to perform statistical analyses. Data were analyzed as descriptive statistics (proportions, percentages, ratios, and frequency distribution tables) and expressed as mean (SD) or as percentages. Bivariate analysis was performed with a *P* value of less than .05 considered significant and was used to establish an association between adherence to hypertension medication and patient-related, drug-related, disease-related, and service-related factors. For bivariate analysis, we categorized frequency of determinants among patients reporting good adherence (MMAS-8 score 7 or 8) and those reporting moderate or nonadherence (MMAS-8 score ≤6) to antihypertension therapy. Multivariate regression analysis was performed to identify independent predictors of adherence by using adherence status as the outcome variable and the other factors as predictor variables.

### Ethical considerations

The study was approved by the ethical committee of the Federal Government Polyclinic. Written informed consent was obtained from each participant before study enrollment following the data collector’s verbal explanation of the study content.

## Results

Our final analysis included 450 of 512 hypertensive patients who fulfilled the study criteria, agreed to participate in the study, and completed questionnaires. Their mean age was 54.5 (SD, 10.6) years (Table 1). Mean body mass index (weight in kg divided by height in m^2^) was 24.1 (SD, 3.9), and 56.2% (253) were male. Good medication adherence was reported by 115 (25.6%) patients; 165 (36.7%) were moderately adherent to medication, and 170 (37.8%) were nonadherent. Almost three-fourths (332 [73.8%]) lived in urban areas. More than two-thirds (303 [67.3%]) did not have a formal education (were illiterate). One hundred twelve (24.9%) were employed in government service. More than half (264 [58.7%]) belonged to the upper lower class on the modified Kuppuswamy scale. None of the responders belonged to the upper socioeconomic status class on the scale. The monthly income of 174 patients (38.7%) ranged from 10,000 to 19,999 Pakistani rupees ($64.50–$129.03). Twenty percent (4.4%) received free medication, of whom 164 (3.6%) reported good adherence, and 4 (0.9%) reported moderate adherence.

**Table 1:** Sociodemographic Characteristics of Patients (N = 450) Diagnosed With Hypertension at a Tertiary Care Hospital, by Level of Adherence^a^ to Medication, Islamabad, Pakistan, 2019

Characteristics	All patients	Nonadherence (n = 170)	Moderate adherence (n = 165)	Good adherence (n = 115)
**Age, mean (SD), y**	54.5 (10.6)	53.4 (11.1)	54.7 (9.8)	55.5 (10.9)
20–39	29 (6.4)	15 (8.8)	8 (4.8)	6 (5.2)
40–59	275 (61.1)	104 (61.2)	100 (60.6)	71 (61.7)
≥60	146 (32.4)	51 (30.0)	57 (34.5)	38 (33.0)
**Male sex**	253 (56.2)	86 (50.6)	89 (53.9)	78 (67.8)
**Marital status**				
Single	6 (1.3)	4 (2.4)	2 (1.2)	0 (0)
Married	403 (89.6)	150 (88.2)	146 (88.5)	107 (93.0)
Divorced or widowed	41 (9.1)	16 (9.4)	17 (10.3)	8 (7.0)
**Residence**				
Urban	332 (73.8)	118 (69.4)	126 (76.4)	88 (76.5)
Rural	118 (26.2)	52 (30.6)	39 (23.6)	27 (23.5)
**Educational status**				
No formal education (illiterate)	303 (67.3)	159 (93.5)	110 (66.7)	34 (29.6)
Literate	147 (32.7)	11 (6.5)	55 (33.3)	81 (70.4)
Primary school or less	29 (6.4)	4 (2.4)	8 (4.8)	17 (14.8)
Up to matriculation or secondary school	77 (17.1)	5 (2.9)	18 (10.9)	54 (47.0)
Bachelor’s degree or higher	41 (9.1)	2 (1.2)	29 (17.6)	10 (8.7)
**Occupation**				
Government employee	112 (24.9)	32 (18.8)	43 (26.1)	37 (32.2)
Housewife	156 (34.7)	59 (34.7)	66 (40.0)	31 (27.0)
Retired	71 (15.8)	25 (14.7)	25 (15.2)	21 (18.3)
Private job	49 (10.9)	21 (12.4)	15 (9.1)	13 (11.3)
Unemployed	25 (5.6)	12 (7.1)	8 (4.8)	5 (4.3)
Laborer or maid	24 (5.3)	17 (10.0)	3 (1.8)	4 (3.5)
Self-employed	13 (2.8)	4 (2.4)	5 (3.0)	4 (3.5)
**Socioeconomic status score^b^**				
Lower (1–4)	91 (20.2)	38 (22.4)	35 (21.2)	18 (15.6)
Upper lower (5–10)	264 (58.7)	104 (61.2)	100 (60.6)	60 (52.2)
Lower middle (11–15)	65 (14.4)	22 (12.9)	16 (9.7)	27 (23.5)
Upper middle (16–25)	30 (6.7)	6 (3.5)	14 (8.5)	10 (8.7)
**Monthly income, rupees ($)**				
<10,000 (64.50)	65 (14.4)	38 (22.4)	17 (10.3)	10 (8.7)
10,000–19,999 (64.50–129.03)	174 (38.7)	71 (41.8)	65 (39.4)	38 (33.0)
20,000–39,999 (129.03–258.06)	179 (39.8)	50 (29.4)	69 (41.8)	60 (52.2)
> 40,000 (>258.06)	25 (5.5)	10 (5.9)	9 (5.5)	6 (5.2)
0	7 (1.6)	1 (0.6)	5 (3.0)	1 (0.9)

Abbreviation: MMAS-8, 8-item Morisky Medication Adherence Scale.

a Level of adherence was classified as good, moderate, or nonadherent according to the MMAS-8; a score of 7 or 8 was classified as good, a score of 6 as moderate, and a score less than 6 as nonadherent. Values are number (percentage) unless otherwise indicated.

b Kuppuswamy scale was used to measure the socioeconomic status of respondents, taking into account a composite score including the education and occupation of the family head and monthly household income, with a score of 1 to 29.

Grade 1 hypertension was diagnosed in 327 (72.7%) patients. The mean duration of hypertension (time from initial diagnosis) was 7.7 (SD, 6.1) years, with 218 (48.4%) of surveyed patients diagnosed in the preceding 5 years (Table 2). A family history of hypertension and ischemic heart diseases was present in 186 (41.3%) patients. One hundred eighty-eight (41.8%) were physically inactive and only 19 (4.2%) were vigorously active with a history of exercise and walking at least 3 times a week. Forty-four (9.8%) patients were current smokers, 53 (11.8%) were former-smokers, and 76 (16.9%) of current smokers had smoked for 5 or more years. The number of patients exposed to passive smoking (secondhand smoke) was 252 (56.0%). The mean distance traveled by patients to reach the health facility was 16.8 km and the mean travel time was 44.9 (SD, 39.5) minutes. More than half of patients (254 [56.4%]) took less than 1 hour to reach the hospital. 

**Table 2:** Distribution of Hypertension-Related Factors Among Patients (N = 450) at a Tertiary Care Hospital, by Adherence^a^ to Medication Regimen, Islamabad, Pakistan, 2019

Characteristics	All patients	Nonadherence (n = 170)	Moderate adherence (n = 165)	Good adherence (n = 115)
**Duration of hypertension, mean (SD), y**	7.7 (SD, 6.1)	7 (SD, 5.9)	8.6 (SD, 6.4)	7.5 (SD, 6.0)
<1	45 (10.0)	21 (12.4)	15 (9.1)	9 (7.8)
1–5	173 (38.4)	75 (44.1)	48 (29.1)	50 (43.5)
6–10	123 (27.3)	37 (21.8)	56 (33.9)	30 (26.1)
11–20	91 (20.2)	33 (19.4)	37 (22.4)	21 (18.3)
≥21	18 (4.0)	4 (2.4)	9 (5.5)	5 (4.3)
**Family history of hypertension and ischemic heart diseases**	186 (41.3)	78 (45.9)	64 (38.8)	44 (38.3)
**Activity level^b^**				
Inactive (<30 min/d)	188 (41.8)	69 (40.6)	74 (44.8)	45 (39.1)
Minimally active (30–60 min/d)	243 (54.0)	97 (57.1)	81 (49.1)	65 (56.5)
Vigorously active (equal to 4 h/d walking)	19 (4.2)	4 (2.4)	10 (6.1)	5 (4.3)
**Smoking status**				
Smoker	44 (9.8)	16 (9.4)	15 (9.1)	13 (11.3)
Never smoked	353 (78.4)	135 (79.4)	130 (78.8)	88 (76.5)
Former smoker	53 (11.8)	19 (11.2)	20 (12.1)	14 (12.2)
Passive smoking	252 (56.0)	99 (58.2)	98 (59.4)	55 (47.8)
**Number of years smoked**				
<5	19 (4.2)	11 (6.5)	6 (3.6)	2 (1.7)
≥5	76 (16.9)	23 (13.5)	29 (17.6)	24 (20.9)
Since childhood (aged 7–10 y)	2 (0.4)	1 (0.6)	0 (0)	1 (0.9)
**Mean distance traveled to reach health facility, km**	16.8 (23.8)	16.1 (22.9)	16.6 (20.3)	16.7 (13.4)
**Mean time to reach health facility, min**	44.9 (39.5)	43.6 (39.7)	47.6 (45.7)	43 (28.5)
≤1 h	254 (56.4)	139 (81.8)	57 (34.5)	58 (50.4)
>1 h	196 (43.6)	31 (18.2)	108 (65.5)	57 (49.6)
**Mean time for a checkup (SD), min**	67.1 (37.7)	73.1 (43.3)	66.8 (35.1)	58.8 (30.6)

a Level of Adherence was classified as good, moderate, or nonadherent according to the MMAS-8; a score of 7 or 8 was classified as good, a score of 6 as moderate, and a score less than 6 as nonadherent. Values are number (percentage) unless otherwise indicated.

b No respondents were in the “Active” category.

The number of patients on a single antihypertension drug was 255 (56.7%); 167 (37.1%) were taking 2 antihypertension medicines, and 28 (6.2%) were taking 3 or more (Table 3). More than half (252 [56.0%]) had a once-daily dosage, and 136 (30.2%) experienced drug-related side effects. Tiredness was the most reported side effect (102 patients, 22.7%), followed by weakness (16 patients, 3.6%,) and dizziness (8 patients, 1.8%). More than half (294 [65.3%]) of participants had concomitant comorbid conditions including diabetes, ischemic heart disease, congestive heart failure, and obesity. Previous hospital admissions for angina, myocardial infarction, stroke, coronary angioplasty, or coronary artery bypass graft were found in 108 patients. The average monthly medication cost was 1,154 Pakistani rupees (US $7.40), and nearly half (223 patients, 49.6%) were not able to afford their monthly medication. The average travel cost to reach a health facility was 125.8 Pakistan rupees (US $0.80). Most (417 [92.7%]) patients were satisfied with their physician’s attitude and care; 419 (93.1%) were satisfied with the prescribed antihypertension drug regimen, and 395 (87.8%) said that counseling was satisfactory. Most respondents (363, 80.7%) had knowledge of hypertension and its prevention and treatment. Almost 91.6% (412) were advised by a physician about the importance of taking hypertensive medication. 

**Table 3:** Clinical and Treatment-Related Characteristics Among Hypertensive Patients (N = 450) at a Tertiary Care Hospital, by Level of Adherence^a^ to Medication, Islamabad, Pakistan, 2019

Characteristics	All patients	Non-adherence (n = 170)	Moderate adherence (n = 165)	Good adherence (n = 115)
**Hypertension duration since therapy, y**				
<1	46 (10.2)	21 (12.4)	15 (9.1)	10 (8.7)
1–5	176 (39.1)	78 (45.9)	49 (29.7)	49 (42.6)
6–10	128 (28.4)	40 (23.5)	58 (35.2)	30 (26.1)
11–20	84 (18.7)	30 (17.6)	33 (20.0)	21 (18.3)
≥21	16 (3.6)	1 (0.6)	10 (6.1)	5 (4.3)
**Number of antihypertension drugs**				
1	255 (56.7)	88 (51.8)	90 (54.5)	77 (67.0)
2	167 (37.1)	70 (41.2)	65 (39.4)	32 (27.8)
≥3	28 (6.2)	12 (7.1)	10 (6.1)	6 (5.2)
**Dosage frequency**				
Once daily	252 (56.0)	102 (60.0)	91 (55.2)	59 (51.3)
Twice daily	198 (44.0)	68 (40.0)	74 (44.8)	56 (48.7)
**Antihypertension medication side effects**				
Yes	136 (30.2)	68 (40.0)	50 (30.3)	18 (15.7)
No	314 (69.8)	102 (60.0)	115 (69.7)	97 (84.3)
**Adverse effects**				
Tiredness	102 (22.7)	51 (30.0)	38 (23.0)	13 (11.3)
Weakness	16 (3.6)	9 (5.3)	5 (3.0)	2 (1.7)
Dizziness and vertigo	8 (1.8)	3 (1.8)	3 (1.8)	2 (1.7)
Headache	5 (1.1)	2 (1.2)	2 (1.2)	1 (0.9)
Stomach pain	4 (0.9)	2 (1.2)	2 (1.2)	0 (0)
Urinary urgency	1 (0.2)	1 (0.6)	0 (0)	0 (0)
**Associated comorbidity**				
Yes	294 (65.3)	114 (67.1)	119 (72.1)	61 (53.0)
No	156 (34.7)	56 (32.9)	46 (27.9)	54 (47.0)
**Type of comorbidity**				
Diabetes	200 (44.4)	72 (42.4)	85 (51.5)	43 (37.4)
Ischemic heart diseases	45 (10.0)	18 (10.6)	18 (10.9)	9 (7.8)
Congestive heart failure	5 (1.1)	0 (0)	2 (1.2)	3 (2.6)
Obesity (BMI ≥30 kg/m^2^)	44 (9.8)	24 (14.1)	14 (8.5)	6 (5.2)
**Hospitalization since diagnosis or in past 2 years if hypertension diagnosed for ≥5 years**				
Yes	108 (24.0)	48 (28.2)	45 (27.3)	15 (13.0)
No	342 (76.0)	122 (71.8)	120 (72.7)	100 (87.0)
**Cause of hospitalization**				
Angina	44 (9.8)	16 (9.4)	24 (14.5)	4 (3.5)
Myocardial infarction	15 (3.3)	12 (7.1)	2 (1.2)	1 (0.9)
Stroke	36 (8.0)	15 (8.8)	16 (9.7)	5 (4.3)
Angioplasty and coronary artery bypass graft	13 (2.9)	5 (2.9)	3 (1.8)	5 (4.3)
**Can afford to purchase monthly medication**				
Yes	227 (50.4)	86 (50.6)	71 (43.0)	70 (60.9)
No	223 (49.6)	84 (49.4)	94 (57.0)	45 (39.1)
**Average monthly cost for hypertension treatment, rupees ($)**	1,154 (7.2)	1,184 (7.4)	1,095 (6.8)	1,183 (7.4)
<1,000 rupees	277 (61.6)	93 (54.7)	95 (57.6)	89 (77.4)
1,000–1,999 rupees	56 (12.4)	37 (21.8)	17 (10.3)	2 (1.7)
2,000–3,999 rupees	31 (6.9)	9 (5.3)	11 (6.7)	11 (9.6)
4,000–4,999 rupees	0 (0)	0 (0)	0 (0)	0 (0)
5,000–8,000 rupees	7 (1.6)	2 (1.2)	2 (1.2)	3 (2.6)
Don’t know/never buy	79 (17.6)	29 (17.1)	40 (24.2)	10 (8.7)
**Average fare to the hospital, rupees**	125.8	130.7	121.6	125.2
**Patient satisfied with physician**	417 (92.7)	151 (88.8)	156 (94.5)	110 (95.7)
**Patient satisfied with prescribed medication**	419 (93.1)	152 (89.4)	157 (95.2)	110 (95.7)
**Agree counseling about hypertension and medication is adequate**	395 (87.8)	142 (83.5)	145 (87.9)	108 (93.9)
**Physician advised about importance of hypertensive medication**	412 (91.6)	154 (90.6)	149 (90.3)	109 (94.8)
**Understands causes and prevention of hypertension**	363 (80.7)	102 (60.0)	154 (93.3)	107 (93.0)
**Reason for not taking antihypertension medication**				
Affordability	73 (16.2)	35 (20.6)	35 (21.2)	3 (2.6)
Lack of access to medicines or health facility	133 (29.6)	57 (33.5)	64 (38.8)	12 (10.4)
Forget to take medicines	41 (9.1)	14 (8.2)	8 (4.8)	19 (16.5)
Do not wish to take medicines	45 (10.0)	10 (5.9)	19 (11.5)	16 (13.9)
Never miss a dose	27 (6.0)	4 (2.4)	14 (8.5)	9 (7.8)
Undesirable effects of medicines	61 (13.6)	29 (17.1)	21 (12.7)	11 (9.6)
Other	70 (15.6)	21 (12.4)	4 (2.4)	45 (39.1)
**Results of noncompliance to medication**				
Acceleration of hypertension symptoms	269 (59.8)	104 (61.2)	100 (60.6)	65 (56.5)
Visit hospital emergency department	100 (22.2)	39 (22.9)	29 (17.6)	32 (27.8)
Absenteeism from work	20 (4.4)	11 (6.5)	7 (4.2)	2 (1.7)
No effect	61 (13.6)	16 (9.4)	29 (17.6)	16 (13.9)

Abbreviations: BMI, body mass index; MMAS-8, 8-item Morisky Medication Adherence Scale.

a Level of adherence was classified as good, moderate, or nonadherent according to the MMAS-8; a score of 7 or 8 was classified as good, a score of 6 as moderate, and a score less than 6 as nonadherent. Values are number (percentage) unless otherwise indicated.

Our bivariate analysis showed that nonadherence was associated with nonaffordability of treatment (OR, 2.54; 95% CI, 1.54–5.03, *P* = .002), uncontrolled hypertension (OR, 3.03; 95% CI, 1.74–5.29, *P* < .001), female sex (OR, 1.44; 95% CI, 1.02–2.96, *P* = .003), and long waiting times in the clinic (OR, 2.93; 95% CI, 1.32–4.84, *P* = .005) (Table 4). Good adherence was associated with satisfactory counseling (OR, 0.31; 95% CI, 0.18–0.54, *P* < .001), the patient’s education (OR, 0.64; 95% CI, 0.41–0.97, *P* = .03), and the presence of comorbidities (OR, 0.62; 95% CI, 0.39–0.94, *P* = .01).

**Table 4:** Bivariate Association Between Hypertension Determinants and Medication Adherence Among Patients (N = 450) in a Tertiary Care Hospital, Islamabad, Pakistan, 2019^a^

Parameter	Frequency in adherent patients (n = 115), MMAS-8 score 8 or 7	Frequency in nonadherent patients (n = 335), MMAS-8 score ≤6	Odds Ratio (95% CI)	*P *value
Treatment not affordable (n = 223)	45 (20.2)	178 (79.8)	2.54 (1.54–5.03)	.002
Uncontrolled hypertension (n = 327)	91 (27.8)	236 (72.2)	3.03 (1.74–5.29)	<.001
Family history (n = 186)	44 (23.7)	142 (76.3)	2.02 (0.57–3.24)	.09
Comorbidities (n = 294)^b^	61 (20.7)	233 (79.3)	0.62 (0.39–0.94)	.01
Education (Yes) (n = 147)	81 (55.1)	66 (44.9)	0.64 (0.41–0.97)	.03
Female sex (n = 197)	37 (18.8)	160 (81.2)	1.44 (1.02–2.96)	.003
Adequate counseling by clinician (n = 395)	108 (27.3)	287 (72.7)	0.31 (0.18–0.54)	<.001
Urban residence (n = 332)	88 (26.5)	244 (73.5)	1.42 (0.93–2.19)	.12
Wait time >60 min for appointment (n = 251)	31 (12.4)	220 (87.6)	2.93 (1.32–4.84)	.005

Abbreviation: MMAS-8, 8-item Morisky Medication Adherence Scale.

a For bivariate analysis, we categorized frequency of determinants among patients reporting good adherence (MMAS-8 score 7 or 8) and those reporting moderate or nonadherence (MMAS-8 score ≤6) to antihypertension therapy. Values are number (percentage) unless otherwise indicated.

b Comorbidites assessed were diabetes, ischemic heart disease, congestive heart failure, and obesity.

After adjusting for age, occupation, sex, residence, and income, multivariate logistic regression showed that nonadherence was associated with unaffordability of treatment (OR, 2.25; 95% CI, 1.46–3.48, *P* = .002) and uncontrolled hypertension (OR, 3.16; 95% CI, 1.76–5.68, *P* < .001). Education (OR, 0.61; 95% CI, 0.39–0.95, *P* = .03) and adequate counseling by clinicians (OR, 0.29; 95% CI, 0.16–0.51, *P* < .001) were associated with good patient adherence (Table 5).

**Table 5:** Multivariate Regression Between Determinants of Medication Adherence Among Patients (N = 450) at a Tertiary Care Hospital, Islamabad, Pakistan, 2019

Parameter^a^	Adjusted odds ratio (95% CI)	*P *value
Treatment unaffordable	2.25 (1.46–3.48)	.002
Uncontrolled hypertension	3.16 (1.76–5.68)	<.001
Education	0.61 (0.39–0.95)	.02
Female sex	1.30 (0.86–1.99)	.20
Urban residence	1.01 (0.63–1.64)	.96
Adequate counseling by clinician	0.29 (0.16–0.51)	<.001
Availability of free blood tests	0.76 (0.39–1.51)	.45

a Parameters adjusted for age, occupation, sex, residence, and income.

## Discussion

To our knowledge, our study is the first to assess determinants of medication adherence in patients previously diagnosed with hypertension in a tertiary care facility in Pakistan. We found a low percentage of hypertensive patients with good adherence to medication regimens. Among patient-related factors, female sex and low education levels were associated with nonadherence. Unaffordability, a lack of access to hypertension medicines, inadequate counseling, and the long waiting times in the clinic were strong predictors of service-related nonadherence. Nonadherence was associated with uncontrolled hypertension, and most patients with elevated blood pressure were prescribed a single drug. The presence of comorbidities was related to better patient adherence.

In our study, the prevalence of good adherence among people in Pakistan with hypertension was lower compared with previous studies in developed countries or other LMICs that found the proportion of patients adherent to antihypertension therapy was between 60% and 90% (12–14). The major regional differences in medication adherence could be related to the health care systems in high-income countries that provide public health facilities, access to universal health care, multisectorial preventive action, adequate care for chronic conditions, and access to affordable antihypertension medication. This accords with a previous study demonstrating that adherence levels and patient economic status are strongly interrelated (14). High medication costs and unaffordable treatment in LMICs are a barrier to accessing health care, leading to a complex circle of nonadherence and resulting complications (14). Given that hypertension is a chronic disease requiring lifelong treatment, affordability becomes a major determinant of adherence for patients in LMICs who lack health insurance or treatment subsidies. Our results support affordability as a determinant of adherence: we found good adherence among patients who either could afford medication or get it free.

Nonadherence is a common phenomenon in uncontrolled hypertension as demonstrated by elevated blood pressure in two-thirds of our study patients. We found that the major reasons for nonadherence to antihypertension medication were lack of access to medicines, including their affordability and availability, long waiting times in the outpatient clinics, and the cost of transportation to visit the clinic. These predictors of nonadherence are modifiable. Strengthening the capacity of clinicians to provide free or low-cost medication can substantially improve adherence, as previously shown in LMICs where patients who reported the availability of free antihypertension medication in the health care facility were nearly twice as likely to be adherent (15).

Unaffordability was unrelated to patient income because our study patients prioritized household expenses over purchasing medicines. The unavailability of antihypertension medication in health care facilities has been reported as a major barrier to health care, particularly in LMICs (16).

Our study also found, as previously reported, that female patients were less likely to be adherent to antihypertension medication (17). The association of poor adherence with female sex illustrates financial, sociocultural, and systemic inequalities that persist despite the concept of universal health care. Gender inequality is a global phenomenon deeply entrenched in many Asian societies (18). It is widely recognized that Pakistani society is dominated by men, with gender gaps in access to all types of resources, especially health care services, supported by a strong regional ideology in some areas that has created a rigid gender hierarchy in society (19). Women are more likely to skip medications, delay treatment, or ask doctors to prescribe low-cost medication because of nonaffordability and inequity (20).

We found that the low education level reported by two-thirds of patients was associated with medication nonadherence. However, although most patients reported hypertension knowledge and most were aware of the importance of taking medications, they did not follow physicians’ recommendations because of other priorities, such as household and family expenses and a lack of resources. Although patient knowledge can improve health outcomes, evaluation of patient information is just one component of a complex intervention. Thus, additional verbal counseling, involvement of family members, or repeated instructions to confirm patient comprehension are key to assisting low-literacy patients when these services are provided by physicians, pharmacists, or nurses (21).

Effective communication skill in medical care can empower patients to engage in disease treatment and is highly correlated with improved adherence to treatment (22). Our study demonstrated that patient satisfaction, effective patient–clinician communication on disease and hypertension therapy, and adequate counseling are important factors associated with good adherence. Ramesh et al showed that comprehensive patient counseling and health education enhanced adherence to therapy among patients with cancer, another noncommunicable disease (23).

Another important barrier to accessing health care services is long waiting times to receive care. Our study provides evidence of a strong association between prolonged waiting time and nonadherence to an antihypertension medication regimen. Several studies have reported that any disease that requires frequent health care visits associated with long waiting times leads to demotivation (24). As indicated in our study, improved and better patient care facilities and reduced waiting time for clinical visits are important factors to increase patient satisfaction and optimize adherence. In this context, LMICs can build health care capacity by improving primary health care and specialist services to ensure universal access to high-quality health care services (25).

Another prominent predictor of nonadherence is medication-related side effects (26). In our study, one-third of patients reported side effects that were unrelated to typical adverse effects of antihypertension drug classes (eg, cough, ankle edema, bradycardia, electrolyte disturbances). Previous studies reported that side effects are the main determinant of low adherence to antihypertension medicines and an important preventive factor in the treatment of hypertension. In one study, 50% of patients discontinued medicines after experiencing side effects (27). The high prevalence of tiredness reported by hypertensive patients in our study may be related to the uncontrolled hypertension itself or the result of lower blood pressure in patients who had high blood pressure for a long time and now need to adjust to lower levels.

In our study, 1 in 10 patients were not willing to take medication, suggesting that patient adherence is likely to increase if the management plan aligns with their preferences. Failure to comply with medication results in wasted medications, disease progression, reduced functional abilities, poor quality of life, and increased use of health care services. These factors impose a heavy economic burden on medical resources and the health care system such as primary care clinics, hospital visits, and hospital admissions. Medication nonadherence leads to poor clinical outcomes and has adverse effects on the health care system. In our study, one-fourth of patients had a history of hospitalization for CVD events, increasing the cost of medical care; also, noncompliance to antihypertension medicines and resultant high blood pressure were associated with accelerated hypertension symptoms in more than half of patients and emergency department visits in one-fourth of patients. The limited resources of the health care system and increased medical costs of hospital visits and admissions are real-time challenges faced by the government and the population of Pakistan. Our results are supported by previous studies examining the association between adherence and risk of hospitalization, medical cost, and its economic impact (28). High medication adherence was associated with lower hospitalization rates, suggesting the importance of improved adherence to reduce the use of health care resources and avoidable health care costs, which can ultimately provide a net economic return by reducing the significant cost burden on health care systems (28).

As in other published studies, our study patients with comorbidities were more adherent to medication. This may be related to other underlying conditions for which patients follow drug regimens based on family history, awareness of CVD risk, more frequent visits to health care facilities, and counseling (29).

### Limitations

Our study had some limitations. First, we included patients from only 1 hospital in Islamabad, a tertiary care facility with a large catchment area. However, Islamabad is Pakistan’s most diverse metropolis, with a diverse population from all ethnicities, and is representative of the country’s population. Ours is the first study to assess numerous determinants associated with medication adherence. Second, although the MMAS-8 questionnaire has been widely used to assess medication adherence, in our study we did not perform other validated methods such as drug urine testing or directly observed pill taking. Other limitations included measuring blood pressure on the right arm and the use of a conventional mercury sphygmomanometer, which is the method recommended in Pakistani hospitals. Our study patients were previously diagnosed with hypertension, and the Pakistan Hypertension Guidelines do not specify in which arm to measure blood pressure at follow-up. Finally, our data were collected before the COVID-19 pandemic, which has had a negative impact on the health care system and the unemployment rate in Pakistan. In our study, the lack of affordability was a predictor of noncompliance; thus, the number of patients with uncontrolled hypertension is likely to increase.

### Conclusion

Our study concluded that three-quarters of hypertensive patients were nonadherent to antihypertension medicines. Unaffordability, female sex, and long waiting times in the hospital health facility were the strongest factors affecting adherence to antihypertension medication among Pakistani patients. The presence of multiple medical conditions, a positive family history of CVD, and a positive patient–clinician relationship through effective counseling were important predictors of better adherence to antihypertension medicines. The availability of free drugs, controlled follow-up visits, and reduced access time to clinical services play an important role in improving adherence. Cost-effective interventions and a multidisciplinary approach directed at the structure of the Pakistani health care system, availability and affordability of blood pressure–lowering medicines, patient education, and adequate patient counseling could improve medication adherence and hypertension management. Our findings may have implications for the implementation of programs on noncommunicable diseases to improve hypertension management and control in Pakistan.
